# Physiologically based pharmacokinetic modeling to predict the pharmacokinetics of codeine in different CYP2D6 phenotypes

**DOI:** 10.3389/fphar.2024.1342515

**Published:** 2024-05-02

**Authors:** Yujie Yang, Xiqian Zhang, Yirong Wang, Heng Xi, Min Xu, Liang Zheng

**Affiliations:** ^1^ Department of Pharmacy, The Third People’s Hospital of Chengdu, College of Medicine, Southwest Jiaotong University, Chengdu, China; ^2^ Department of Clinical Pharmacology, The Second Affiliated Hospital of Anhui Medical University, Hefei, China

**Keywords:** codeine, physiologically based pharmacokinetic, CYP2D6, genetic polymorphism, pharmacokinetics

## Abstract

**Objectives:**

Codeine, a prodrug used as an opioid agonist, is metabolized to the active product morphine by CYP2D6. This study aimed to establish physiologically based pharmacokinetic (PBPK) models of codeine and morphine and explore the influence of CYP2D6 genetic polymorphisms on the pharmacokinetics of codeine and morphine.

**Methods:**

An initial PBPK modeling of codeine in healthy adults was established using PK-Sim^®^ software and subsequently extrapolated to CYP2D6 phenotype-related PBPK modeling based on the turnover frequency (K_cat_) of CYP2D6 for different phenotype populations (UM, EM, IM, and PM). The mean fold error (MFE) and geometric mean fold error (GMFE) methods were used to compare the differences between the predicted and observed values of the pharmacokinetic parameters to evaluate the accuracy of PBPK modeling. The validated models were then used to support dose safety for different CYP2D6 phenotypes.

**Results:**

The developed and validated CYP2D6 phenotype-related PBPK model successfully predicted codeine and morphine dispositions in different CYP2D6 phenotypes. Compared with EMs, the predicted AUC_0-∞_ value of morphine was 98.6% lower in PMs, 60.84% lower in IMs, and 73.43% higher in UMs. Morphine plasma exposure in IMs administered 80 mg of codeine was roughly comparable to that in EMs administered 30 mg of codeine. CYP2D6 UMs may start dose titration to achieve an optimal individual regimen and avoid a single dose of over 20 mg. Codeine should not be used in PMs for pain relief, considering its insufficient efficacy.

**Conclusion:**

PBPK modeling can be applied to explore the dosing safety of codeine and can be helpful in predicting the effect of CYP2D6 genetic polymorphisms on drug–drug interactions (DDIs) with codeine in the future.

## 1 Introduction

Codeine is a relatively selective opioid agonist for the mu-opioid receptor but has a much weaker affinity than morphine (Thorn et al., 2009). Of these, approximately 70%–80% of codeine is metabolized by *UDP*-glucuronyl transferase (UGT) 2B7 and 2B4 to form codeine-6-glucuronide, and 5%–15% of codeine is N-demethylated by cytochrome P450 3A4 (CYP3A4) to produce demethylcodeine. A small percentage of demethylcodeine was further O-demethylated to form demethylmorphine. The most crucial metabolism of codeine occurs via cytochrome P450 2D6 (CYP2D6), and CYP2D6 demethylates 5%–10% of codeine to form the active metabolite morphine. Approximately 60% of morphine is converted to inactive morphine-3-glucuronide (M3G) and approximately 10% to active morphine-6-glucuronide (M6G) in the liver, mainly by UGT (FDA, 2023). Morphine and M6G have opioid activities, and the analgesic properties of codeine have been speculated to originate from its conversion to morphine. CYP2D6 is a crucial factor for the curative effect of codeine *in vivo*.

CYP2D6 is a highly polymorphic gene, with many crucial single-nucleotide polymorphisms, haplotypes, and copy number variants (Gough et al., 1993). CYP2D6 genetic polymorphisms can lead to differences in enzyme activity, directly affecting drug exposure *in vivo*. According to the different enzymatic activities, CYP2D6 variants can be categorized into four phenotypes as follows: extensive metabolizer (EM), intermediate metabolizer (IM), poor metabolizer (PM), and ultra-rapid metabolizer (UM) (Somogyi et al., 2007). Pharmacokinetic (PK) studies have shown significant differences in the drug exposure of the codeine metabolite morphine in populations with different CYP2D6 gene phenotypes (Linares et al., 2015). Genetic polymorphisms in CYP2D6 are associated with diminished pain relief or severe side effects. However, the Food and Drug Administration (FDA) has not published recommendations for the administration schedule adjustment of codeine in populations with different CYP2D6 phenotypes. Therefore, effective methods are needed to provide reliable evidence for the clinical application of codeine in different phenotypes.

Physiologically based pharmacokinetic (PBPK) modeling can simulate the process of drug disposal in the body based on anatomy, physiology, biochemistry, and physical chemistry, where tissues or organs are connected by blood circulation (Grimstein et al., 2019). The essential components of the PBPK model include drug- and non-drug-dependent physiologies. Non-drug-dependent physiology includes the abundance and activity of drug transporters and metabolic enzymes. With the development of PBPK software and human genomics in recent years, pharmacogenomics has provided new ideas for personalized medicine with the help of PBPK modeling. Previous studies have developed CYP2D6 phenotype-related PBPK models to explore the effect of CYP2D6 gene polymorphisms on pharmacokinetics (Grzegorzewski et al., 2022) and elucidate the extent of CYP2D6-mediated drug–drug interactions (DDIs) (Dickschen et al., 2017; Xu et al., 2021). PBPK modeling can reduce unnecessary clinical trials and maximize existing data. We used data extrapolation to improve and enrich drug use information for populations with different CYP2D6 gene phenotypes.

Several studies have used the PBPK model to explore the effects of ontogeny and genetic variation of organic cation transporter 1 or UGT2B7 on morphine disposal (Emoto et al., 2018; Schaefer et al., 2018; Sjöstedt et al., 2021). However PBPK modeling has not been established to explore the dose safety of codeine for different CYP2D6 phenotypes. Our study aimed to develop a CYP2D6 phenotype-related PBPK model of codeine and morphine and provide a scientific basis for codeine pharmacokinetics in different CYP2D6 phenotypes. This study also aimed to expand the knowledge field of CYP2D6 drug–gene interactions and DDI scenarios.

## 2 Methods

### 2.1 PBPK modeling development workflow and computer software

**FIGURE 1:**
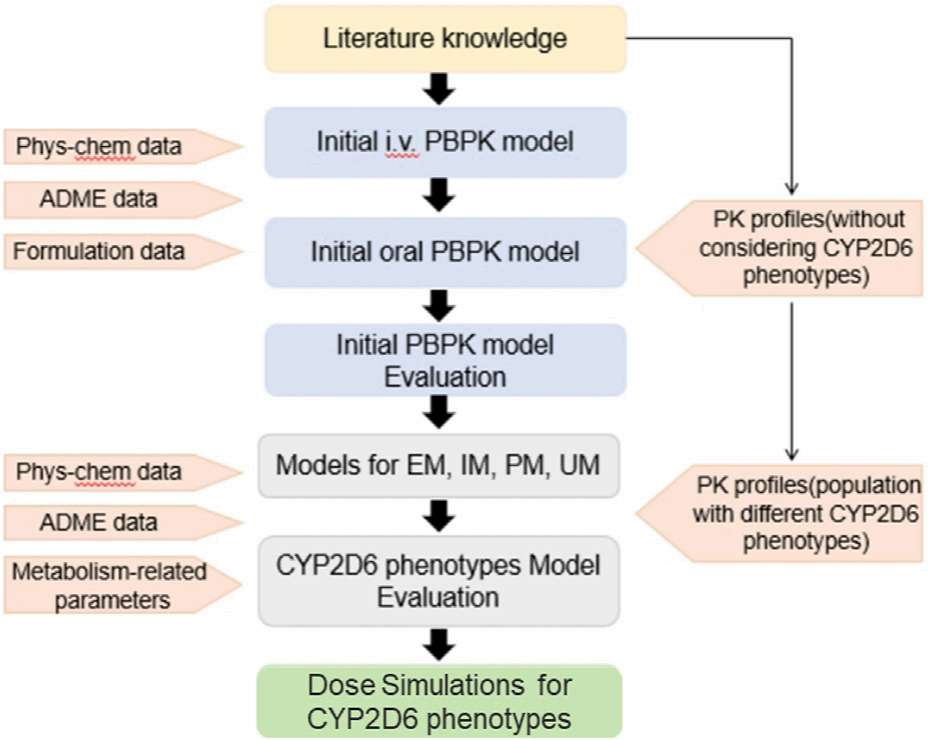
Schematic representation of the workflow of PBPK modeling development.

This study used a ‘bottom-up’ strategy to facilitate the PBPK modeling of codeine and morphine (Figure 1). First, we developed initial PBPK models of codeine and morphine without considering CYP2D6 phenotypes. Subsequently, CYP2D6 phenotype-related PBPK modeling was developed based on the initial model.

The physicochemical and ADME properties of codeine and morphine and reported PK profiles following i. v. and p. o. administration of codeine were obtained by searching PubMed, Web of Science, the MEDLINE database, or the “DrugBank” database using the keywords ‘codeine,’ ‘morphine,’ and ‘pharmacokinetics.’ The particular keywords for adults with different CYP2D6 phenotypes were ‘CYP2D6,’ ‘phenotype,’ ‘genotype,’ and ‘metabolizers.’ The collected literature was organized according to the experimental data (plasma concentration–time profile) and dosing regimen, and we excluded studies on the determination of codeine and morphine plasma concentrations using the microbial method (Supplementary Table S1).

All simulations were performed using PK-Sim^®^ (version 11.2.0). Plasma concentration–time profile data in the published literature were obtained using the GetData Graph Digitizer (S. Fedorov, version 2.25.0.32). The observed PK parameters were obtained from the literature or calculated using non-compartment analysis by Phoenix software (version 8.1.0). Graphics were created and edited using GraphPad Prism (version 6.02).

### 2.2 Codeine and morphine PBPK modeling development

The PBPK model, comprising 18 tissue/organ compartments, was developed using PK-Sim^®^ model software, where human physiological parameters were obtained using the virtual population. The physicochemical and ADME parameters of codeine and morphine are shown in Table 1. Codeine is metabolized in the liver mainly by CYP2D6 (morphine), CYP3A4 (demethylcodeine), and UGT2B7 (codeine-6-glucuronide, C6G). The relevant enzyme kinetic parameters (K_m_ and K_cat_) were optimized using parameter identification with the built-in Monte Carlo algorithm by fitting to observed PK data. Parameter identification is a means of parameter optimization to minimize the residuals between observed PK data and corresponding simulation output (codeine and morphine blood concentration) by changing the selected input parameters (Km and Kcat) in a given range. Renal clearance (CLR) combines the glomerular filtration rate (GFR), tubular secretion, and reabsorption. The total CLR was corrected according to Susanne, which met the observed value of the fraction excreted unchanged via the kidney. UGT2B7 can further metabolize morphine to form glucuronides. Liver clearance is characterized by total liver clearance, and CLR is optimized using DrugBank data. In addition, the PK-Sim^®^ standard method was used to estimate cellular permeability, and the Rodgers and Rowland method was used for determining the partition coefficient.

**TABLE 1:** Summary of input compound parameters of codeine and morphine.

Parameter	Codeine		Morphine	
	Value	Source/method	Value	Source/method
*Physico-chemical*				
logP	2.6	DrugBank	0.77	DrugBank
pKa-acid	13.78	ChemAxon	8.21	DrugBank
pKa-basic	9.19	ChemAxon	7.93	DrugBank
MW, g/mol	299.36	DrugBank	285.34	DrugBank
Solubility at pH7, mg/mL	0.577	ALOGPS	10.2	ALOGPS
fup	0.925	Vree and Verwey-Van Wissen (1992)	0.654	Sjöstedt et al. (2021)
Blood to plasma ratio (B/P)	0.97	Xie and Hammarlund-Udenaes (1998)	1.34	Mistry and Houston (1987)
*ADME*				
Partition coefficients	Rodgers and Rowland		Rodgers and Rowland	
Cellular permeabilities	PK-Sim^®^ standard		PK-Sim^®^ standard	
K_m, 3A4_ (μM)	104.1	Parameter identification		
K_cat, 3A4_ (/min)	2.8	Parameter identification		
K_m, 2D6_ (μM)	100	Parameter identification		
K_cat, 2D6_ (/min)-EM	1.6	Parameter identification		
K_m, UGT2B7_ (mM)	2.32	Raungrut et al. (2010)	0.212	Mistry and Houston (1987)
V_max, UGT2B7_ (pmol/min/mg)	573	Raungrut et al. (2010)	650	Mistry and Houston (1987)
CL_met_ (mL/min/kg)	20.32	Ammon et al. (2002)		
CL_renal_ (mL/min/kg)	5	Guay et al. (1987)	6.5	DrugBank
Specific CL (/min)	1.88	Parameter identification	5.14	Parameter identification
*Formulations*				
Dissolution time (C) (min)	5	Parameter identification		
Dissolution shape (C)	1	Parameter identification		
Dissolution time (T) (min)	5	Parameter identification		
Dissolution shape (T)	0.92	Parameter identification		
Dissolution time (SR) (min)	100	Parameter identification		
Dissolution shape (SR)	1.2	Parameter identification		

LogP, lipophilicity; PKa, dissociation constant (acid and basic); fuP, fraction unbound; B/P: blood to plasma concentration ratio; K_m_, Michaelis constant; V_max_, maximum enzyme reaction rate; k_cat_, turnover frequency of enzyme; CL_met_, total hepatic clearance; CL_renal_, renal clearance; specific CL, is calculated from plasma clearance as input value; dissolution time, 50% dissolved; C, capsule; T, tablets; SR, sustained-release (SR) tablets.

Oral formulations included immediate-release (IR) tablets, sustained-release (SR) tablets, control-release (CR) solutions, and ordinary tablets/capsules/solutions. The IR tablet formulation was set as dissolved. The release features of the uncoated tablet/capsule and SR tablets were described using the Weibull function. The different dosage forms of codeine were calculated and converted into the corresponding administration regimens according to the content of codeine.

### 2.3 CYP2D6 phenotype-related PBPK modeling development

Based on the model of codeine and morphine without considering the genotype, the physicochemical properties of all drug parameters (molecular weight, log P, or Kp values) were fixed, and the genotype activity value of CYP2D6 was used to determine the phenotype of CYP2D6. CYP2D6 genotyping was conducted based on the activity score (AS) of the different CYP2D6 alleles divided into EM, IM, PM, and UM types (Supplementary Table S2). The turnover frequency (K_cat_) of the EM type with an AS value of 1.5 was used as a reference (100%) and set to 1.6/min, and K_cat_ of other phenotypes was calculated according to the ratio of the AS value. K_cat_ of the CYP2D6 IM type was calculated as the median value of PM and EM and verified by clinical studies (Table 2). To avoid affecting the calculation, K_cat_ of the CYP2D6 PM type was not directly set to 0. The Monte Carlo method used the EM, PM, IM, and UM clinical data to optimize K_cat_ of CYP2D6.

**TABLE 2:** Optimized K_cat, rel_ values for the different modeled CYP2D6 activity scores.

Activity score	Phenotype	K_cat_, _CYP2D6_ (/min)	k_cat, rel_ ^a (%)^	Literature
0	PM	0.03	2	Kirchheiner et al. (2007)
0.5	IM	0.40	25	Wu et al. (2014)
0.75	IM	0.6	37.5	
1	IM^b^	0.8	50	Tseng et al. (1996)
1.25	EM	0.96	65	(Wu et al., 2014; Yue et al., 1991a; Mikus et al., 1997; Kronstrand and Jones, 2001; Eckhardt et al., 1998)
1.5	EM	1.6	100	
1.75	EM	1.87	117	
2	EM	2.16	135	(Kirchheiner et al., 2007; Chen et al., 1991; Tseng et al., 1996)
2.5	UM	2.4	150	Wu et al. (2014)
3	UM	2.8	175	Kirchheiner et al. (2007)

^a^ The catalytic rate constant was relative to activity score = 1.5.

^b^ K_cat_ of the CYP2D6 IM type was calculated as the median value of PM and EM.

EM, extensive metabolizer; IM, intermediate metabolize; PM, poor metabolizer; UM, ultra-rapid metabolizer.

### 2.4 PBPK modeling evaluation

For the PBPK model evaluation, we used virtual populations generated by software with the same ethnic and demographic information as in the original literature, and the model performance was evaluated using different methods. We visually inspected the simulated and observed data following various dosing regimens. In addition, the goodness-of-fit of the plasma drug concentration and PK parameters were calculated. The PK parameters of the observed values were directly obtained from geometric or arithmetic means reported in the literature. If no PK parameters were reported, the intercepted plasma drug concentration values were used for non-compartment analysis. The mean fold error (MFE) and geometric mean fold error (GMFE) methods were used to compare the differences between the predicted and observed values of the PK parameters to evaluate the accuracy of the PBPK model. When the MFE and GMFE of PK parameters were within a 0.5- to 2-fold error (Sager et al., 2015) range, we considered the PBPK model establishment successfully.

### 2.5 Codeine dose simulation

We simulated codeine doses in different genotypes using genotype-dependent CYP2D6-mediated clearances of codeine. First, we analyzed the predictions of codeine and morphine in different populations with different CYP2D6 phenotypes under an administration regimen of 30 mg of codeine tablets. Then, we used the established PBPK models to simulate the situation in Asians with different CYP2D6 phenotypes after different doses. To intuitively compare different CYP2D6 phenotypes, we used the AS median value of each phenotype as a representative. All simulations were investigated in 1,000 virtual patients.

## 3 Results

### 3.1 Healthy adult PBPK modeling establishment and evaluation

**FIGURE 2:**
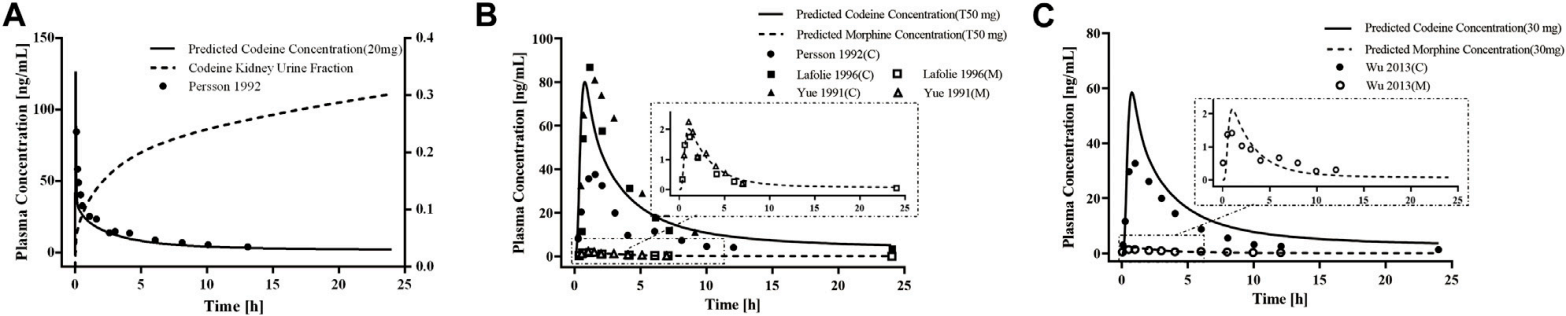
PBPK modeling established in healthy adults. Simulation (lines) of PK profiles for a single **(A)** i.v. administration of 20 mg of codeine and **(B)** p. o. administration of 50 mg of codeine tablet in a typical European individual, **(C)** p. o. administration of 30 mg of codeine tablet in a typical Asian individual. The observed concentration data were provided as the arithmetic mean values extracted from references. The solid and dotted lines represented the predicted codeine and morphine concentrations, respectively.

First, we developed a PBPK model of codeine i. v. administration based on a typical 30-year-old European male individual with average physiological parameters. We found that using the fractions of excreted urine unchanged was consistent with the observed data obtained from the European adult population (Figure 2). We then developed the PBPK model of codeine p. o. administration based on a typical 30-year-old European and Asian male individual. Parameters were randomly optimized to obtain the best-fit parameters using the observed values from four clinical studies. The established adult codeine and morphine PBPK models are shown in Figure 2. GMFEs of AUC_0-∞_ and C_max_ in established adult codeine, unconjugated codeine, and morphine PBPK models are shown in Supplementary Tables S3, S4.

**FIGURE 3:**
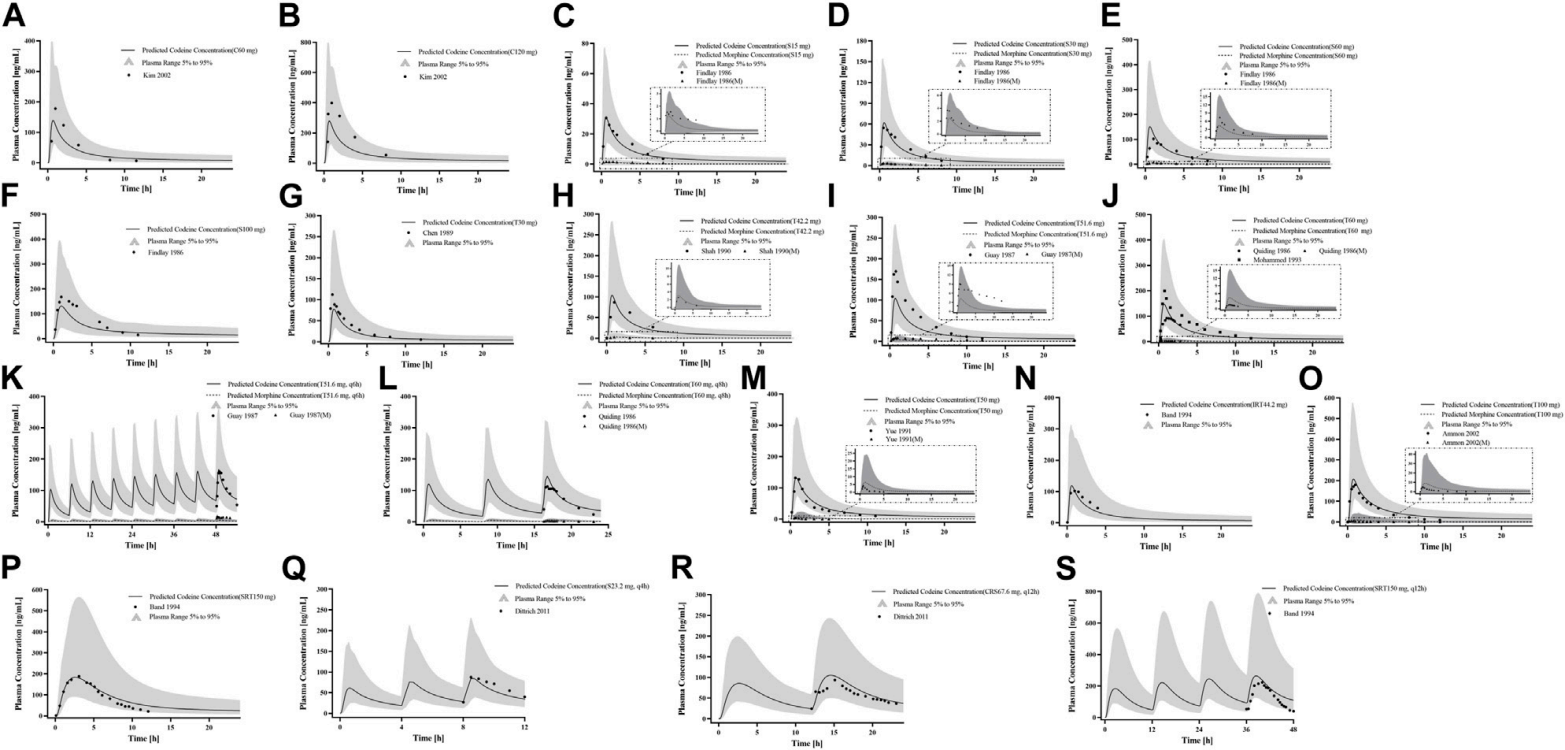
PK profile verification in healthy adults. **(A, B)** Single p. o. administration of codeine capsules in African Americans; **(C–L)** the p. o. administration at a dose or multiple doses of codeine in different formulations in White Americans; **(M)** the single p. o. administration of 50 mg codeine tablets in Asians; and **(N–S)** the p. o. administration at a dose or multiple doses of codeine in different formulations in Europeans. The observed concentration data were provided as the arithmetic mean values extracted from references. The solid and dotted lines represented the predicted codeine and morphine concentrations, respectively. The shaded area represented the predicted 5th to 95th percentile range.

**FIGURE 4:**
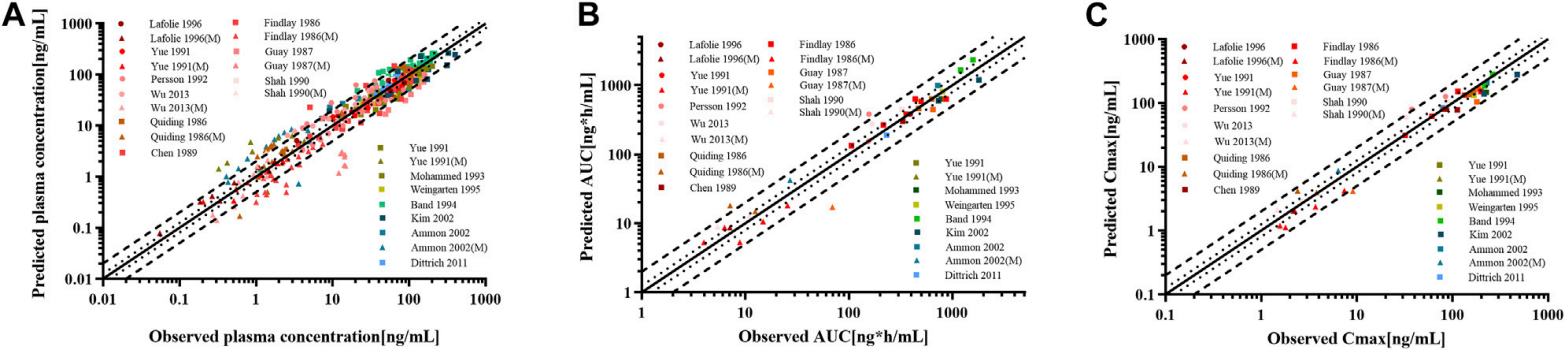
Goodness-of-fit plot for **(A)** plasma concentrations, **(B)** AUC_0-∞_, and **(C)** C_max_ of codeine and morphine in the healthy adult population. The solid line represented the line of identity, and the bold dotted and dotted lines represented the 2-fold and 1.25-fold error ranges, respectively. The circles and squares indicated data used to PBPK modeling, building, and evaluation, and the triangle showed data about morphine.

For the healthy adult PBPK model evaluation, 11 clinical studies following various dosing regimens were identified and used (Figure 3). For the model evaluation, models with single dosages of capsules, solutions, tablets, and sustained-release tablets and multiple dosages of tablets, sustained-release tablets, and control-release solutions were built. The goodness-of-fit diagram of codeine plasma drug concentrations showed that more than 89.11% of the predicted drug concentration values were within a 2-fold error range of the observed values, and approximately 68.09% of the predicted values were within a 1.5-fold margin of error (Figure 4). The metabolite morphine was not as well-fitted as codeine, with only 63.25% of the plasma drug concentrations within the 2-fold range. The MFE average and GMFE of all predicted PK parameters were within the 1.5-fold error range in both codeine and morphine PBPK models (Supplementary Table S4), except for the GMFE of AUC_0-∞_ in morphine PBPK models.

### 3.2 CYP2D6 gene-related PBPK modeling establishment and evaluation

**FIGURE 5:**
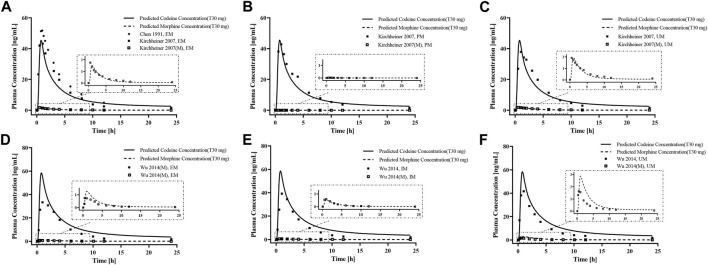
PBPK modeling established in healthy adults with different CYP2D6 phenotypes. Simulation (lines) of PK profiles for a single p. o. administration of 30 mg codeine tablet in a typical American individual with **(A)** EM type, **(B)** PM type, and **(C)** UM type, and p. o. administration of 30 mg codeine tablet in a typical Asian individual with **(D)** EM type, **(E)** IM type, and **(F)** UM type. The observed concentration data were provided as the arithmetic mean values extracted from references. The solid and dotted line represented the predicted codeine and morphine concentrations, respectively.

**FIGURE 6:**
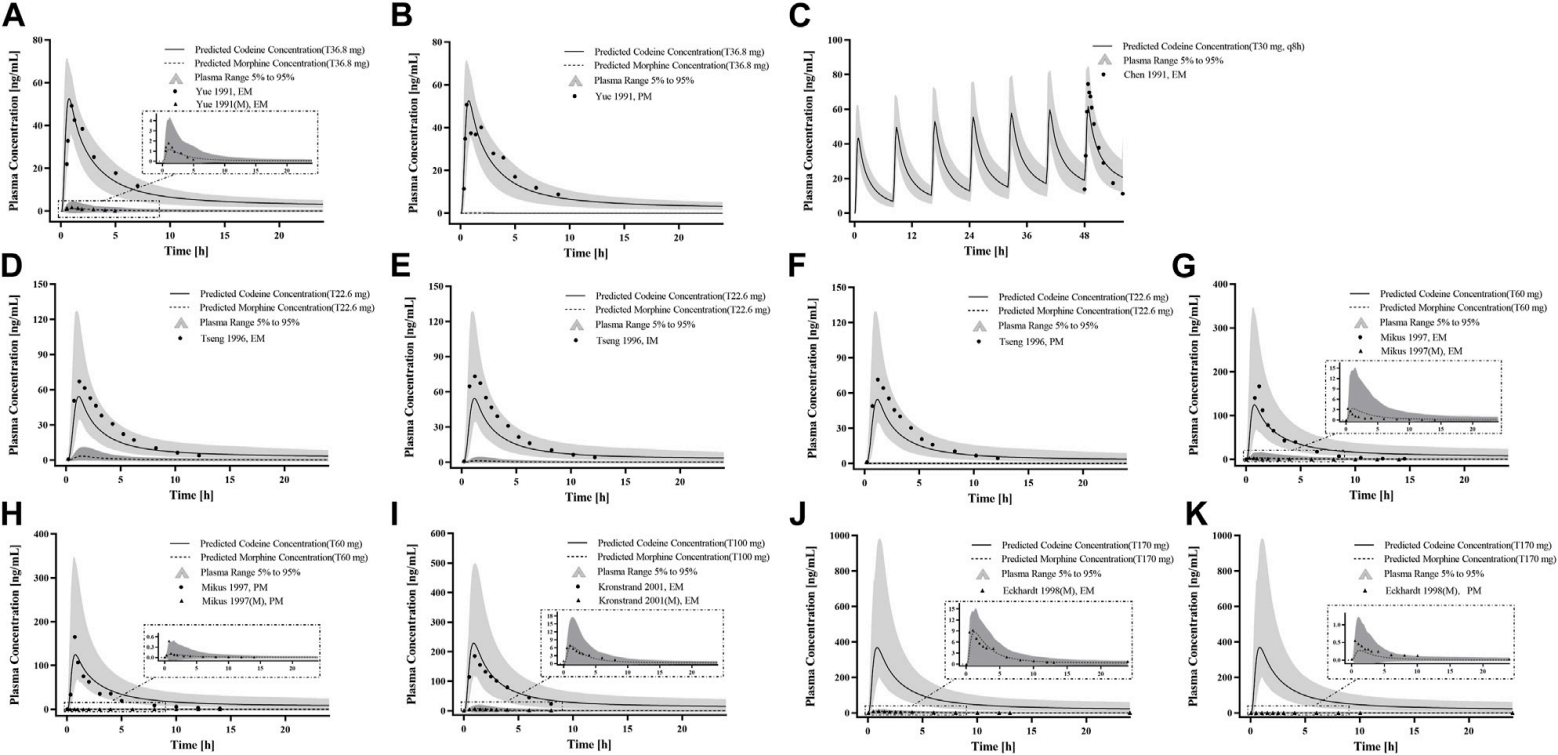
PK profile verification in adults with different CYP2D6 phenotypes. **(A–C)** p. o. administration at a dose or multiple doses of codeine tablets in Americans with EM and PM types; **(D–F)** the single p. o. administration of codeine tablets in Asians with EM, IM, and PM types; and **(G–K)** the single p. o. administration of codeine tablets in Europeans with EM and PM types. The observed concentration data were provided as the arithmetic mean values extracted from references. The solid line and dotted line represented the predicted codeine and morphine concentrations, respectively. The shaded area represented the predicted 5th to 95th percentile range.

First, we predicted the PK of codeine and morphine in healthy adults with different CYP2D6 phenotypes using the physicochemical properties of the drugs along with different K_cat_ values of CYP2D6 (Figure 5). We found that the single AS value corresponding to the genotype could not simulate the level of morphine *in vivo* well; therefore, we further divided the range of AS values (Table 2). For the CYP2D6 gene-related model of codeine and morphine, eight clinical studies were used for model establishment and evaluation. We set K_cat_ of PM to 0.03/min according to the parameter identification. On visual inspection, the AS values of 0.5 and 1 fitted Wu et al. (2014) and Tseng et al. (1996) in IMs, and the AS values of 2.5 and 3 fitted Wu et al. (2014) and Kirchheiner et al. (2007) in UMs, respectively. As for the EMs, the AS value of 1.25 matched Wu et al. (2014), Yue et al. (1991a), Mikus et al. (1997), Kronstrand and Jones (2001), and Eckhardt et al. (1998), and the AS value of 2 matched Kirchheiner et al. (2007), Chen et al. (1991), and Tseng et al. (1996) (Figure 6).

**FIGURE 7:**
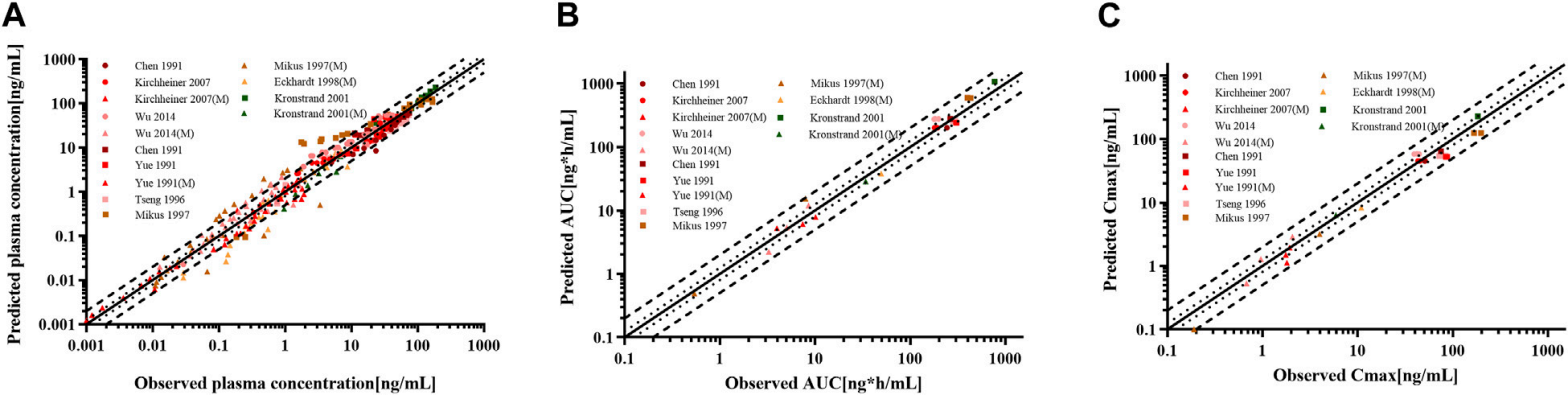
Goodness-of-fit plot for **(A)** plasma concentrations, **(B)** AUC_0-∞_, and **(C)** C_max_ of codeine and morphine in adults with different CYP2D6 phenotypes. The solid line represented the line of identity, and the bold dotted lines and dotted lines represented the 2-fold and 1.25-fold error ranges, respectively. The circles and squares indicated data used for PBPK modeling, building, and evaluation, and the triangle showed data about morphine.

The goodness-of-fit diagram of morphine plasma drug concentrations showed that more than 76.92% of the predicted drug concentration values were within the 2-fold error range of the observed values, and approximately 53.85% were within 1.5-fold (Figure 7). The results showed that the simulation of the metabolite morphine was better after distinguishing the CYP2D6 phenotypes. The average MFE and GMFE of AUC_0-∞_ and C_max_ were within a 1.5-fold error range in EMs, IMs, and UMs (Supplementary Table S4). Due to the low concentration of morphine in PMs, the deviations of morphine tended to have larger GMEF values (1.45 and 1.84 for AUC_0-∞_ and C_max_), but this deviation was not clinically significant.

### 3.3 Codeine dose simulation based on CYP2D6 genetic polymorphism

**FIGURE 8:**
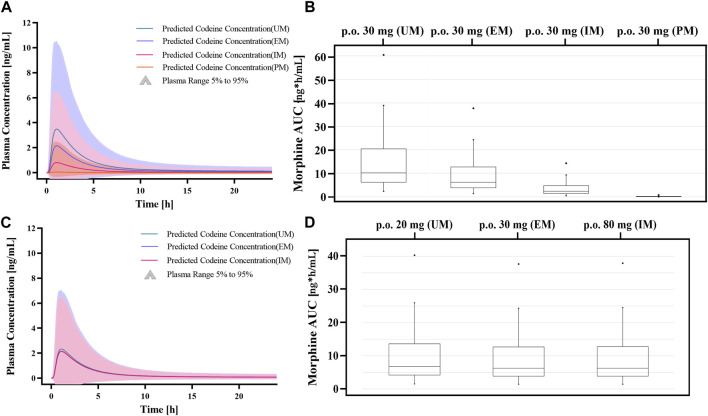
Dose simulations based on CYP2D6 gene-related PBPK modeling. **(A, B)** Simulations of morphine exposure and AUC_0-∞_ in Asians with different CYP2D6 phenotypes at administration of a 30 mg codeine tablet. **(C, D)** Simulations of morphine exposure and AUC_0-∞_ in Asians with different CYP2D6 phenotypes with the model-based dose.

Genetic polymorphisms in CYP2D6 are associated with diminished pain relief or severe side effects. Compared with EMs, the predicted AUC_0-∞_ of morphine was 98.6% lower in PMs, 60.84% lower in IMs, and 73.43% higher in UMs under an administration regimen of 30 mg codeine tablets (Supplementary Table S5). The results showed that the errors were controlled below 30%, except for codeine in the IMs. In addition, box–whisker and time-profile analyses were used for direct comparisons in Asians with different CYP2D6 phenotypes (Figure 8). As shown in Table 3, a dose or multiple doses of 80 mg of codeine in IMs, 1500 mg in PMs, and 20 mg in UMs had an approximately equivalent plasma exposure of morphine in EMs administered 30 mg of codeine. Notably, the simulated PM dose exceeded the daily maximum amount of codeine. Alternative analgesics are recommended for PMs who do not perform well on codeine.

**TABLE 3:** Simulated pharmacokinetic parameters of morphine in Asians with different CYP2D6 phenotypes.

Phenotypes	EM (AS = 1.5)	IM (AS = 0.75)	PM (AS = 0)	UM (AS = 2.75)
Single dose				
Dose	30 mg	80 mg	1500 mg	20 mg
AUC (ng·h/mL)	9.07	9.1	7.34	9.75
Cmax (ng/mL)	2.16	2.15	1.41	2.34
Tmax h)	1.05	1.05	1.05	1.05
Multiple dose				
Dose	30 mg	80 mg	1500	20 mg
AUC (ng·h/mL)	11.55	11.61	8.95	12.40
Cmax (ng/mL)	2.83	2.82	1.91	3.05

EM, extensive metabolizer; IM: intermediate metabolize, PM, poor metabolizer, UM, ultra-rapid metabolizer.

## 4 Discussion

Our study successfully developed a PBPK model of codeine and its active metabolite (morphine). Based on the verified basic model of codeine and morphine, the enzyme kinetic parameters of CYP2D6 with different gene phenotypes were optimized using the Monte Carlo method. The CYP2D6 gene-related PBPK modeling was successfully established using the PK data of clinical populations with different CYP2D6 gene phenotypes as observed values. The critical point in the establishment of PBPK modeling of codeine based on CYP2D6 genetic polymorphism is the change in enzyme kinetic parameters of different CYP2D6 gene phenotypes. K_cat_ of CYP2D6 can represent the catalytic efficiency, defined as the number of substrate molecules converted into a product at each enzyme site per unit of time. It was important to note that when we developed an initial PBPK model of codeine and morphine without considering CYP2D6 phenotypes, we set the K_cat_ rate of CYP2D6 to 1.6/min, precisely the median in the EMs. Therefore, the prediction of morphine in the initial PBPK modeling was poor, and the GMFE of AUC_0-∞_ in the morphine PBPK model was high. In addition, when we further adjusted the K_cat_ rate according to CYP2D6 phenotypes, we found that a single AS value corresponding to the CYP2D6 phenotypes could not accurately simulate the level of morphine in each literature work. So, we further divided the range of the AS value, where IMs included three levels of 0.5-1, EMs included four levels of 1.25–2, and UMs included two levels of 1.25–2. Notably, K_cat_ of CYP2D6 in the PM type was set to 0 in the previous model building (Chen et al., 2018). That is, the metabolic capacity of CYP2D6 in the PM type is entirely lost. However, previous clinical PK studies have shown that morphine generated by codeine via CYP2D6 was still exposed to CYP2D6 PMs (Kirchheiner et al., 2007). In this study, K_cat_ of CYP2D6 PM type was not set to 0 directly, and we set K_cat_ of PM to 0.03/min according to clinical PK studies of codeine and morphine in PMs (Kirchheiner et al., 2007).

In our predictions, the plasma exposure of morphine in IMs and PMs was significantly lower than that in EMs, which was consistent with observations in published studies. In our study, compared with EMs, the predicted AUC_0-∞_ value of morphine was 98.6% lower in PMs, 60.84% lower in IMs, and 73.43% higher in UMs. Notably, the adverse events related to morphine-related deaths were reported to be associated with ultrafast codeine metabolism (Ciszkowski et al., 2009). The FDA drug descriptions stated that the UMs converted codeine into its active metabolite, morphine, more rapidly and completely, resulting in higher serum morphine levels. Even with labeled dosage regimens or simulated dosages, UMs may have life-threatening or fatal respiratory depression or may experience signs of overdose (FDA, 2023). In addition, we found that the simulated dosage of PMs exceeded the daily maximum amount of codeine. The labeled dose of codeine may be unable to be efficiently converted to morphine. In summary, codeine should not be used by PMs for pain relief considering its insufficient efficacy; therefore, other drugs should be considered for analgesic sedation. For IMs, our predictions showed that the dose of codeine increased by a factor of 1.67 and had an approximately equivalent morphine plasma exposure as in EMs. The prevalence of CYP2D6 phenotypes varies widely. PMs and UMs have been estimated to be 5%–10% and 1%–10% for Caucasians (European and North American), 0%–19% and 3%–4% for Black people (African Americans) (Mendoza et al., 2001), respectively, and both 1% and 2% for Asians (Chinese, Japanese, and Korean) (Bradford, 2002). Additionally, UMs may be greater than 10% in certain racial/ethnic groups (that is, the Middle East) (Khalaj et al., 2019). An “a priori” and correct determination of CYP2D6 phenotypes, combined with our PBPK modeling, can promise individual predictability in drug exposure to morphine be realized.

The clinical implications of this study require further discussion. The medical indications for codeine include cough suppression and analgesia. The antitussive activity of codeine is approximately 1/4 that of morphine, and its analgesic effect is approximately 1/10 that of morphine (Beaver et al., 1978). However, the exposure of codeine after administration is much higher than that of morphine (>20 folds). Although the accurate relative contributions to *in vivo* activity have not been determined, evidence-based data have indicated the necessity to adjust the regimen according to the CYP2D6 genotype. The Clinical Pharmacogenetics Implementation Consortium (CPIC) and the Dutch Pharmacogenetics Working Group (DPWG) both recommend avoiding codeine in individuals identified as CYP2D6 PMs for pain control due to significantly reduced morphine formation and insufficient pain relief (Pratt et al., 2012). This study confirms the recommendation by indicating that a markedly excessive dose of codeine is needed in PMs to obtain similar exposure to morphine. On the other hand, the DPWG recommends that no actions be required in IMs and PMs for cough prevention (Matic et al., 2022). This reflects the organization’s opinion that the antitussive activity comes primarily from the parent compound. According to the FDA’s codeine medication label, UMs may develop symptoms of overdose or potentially deadly respiratory depression, even at prescribed dosages (FDA, 2017). Although the FDA suggests that CYP2D6 UMs should not use codeine tablets, the DPWG is considered a lower dose acceptable (Matic et al., 2022). According to our simulation, a 1/3 dose reduction in UMs compared to EMs is appropriately measured by morphine formation, while the discrepancy in codeine exposure is minimal. In practice, CYP2D6 UMs may start dose titration to achieve an optimal individual regimen and avoid a single dose over 20 mg. With accumulated phenotype-based efficacy evidence, the models developed in this study can be easily used to formulate dose adjustments.

We acknowledge that this study has limitations. We did not perform pharmacodynamics simulations to determine the effects of CYP2D6 genetic polymorphisms on codeine efficacy (separately for pain control and cough prevention). Relying only on pharmacokinetic information to guide the individual administration of a drug has limitations to some extent. This study also did not address the drug evaluation at extremes of age, and we did not distinguish the difference in CYP2D6 activity between different ethnic groups in the healthy adult models. More studies are needed to explore the pharmacodynamic modeling of codeine in the future, especially the effects of genetic polymorphisms in specific populations. Our study provided a theoretical basis for exploring CYP2D6-mediated codeine interactions with other drugs. For more accurate DDI scenarios, the enzyme kinetic parameters for both codeine and inducer/inhibitors should be explored.

## 5 Conclusion

We developed and validated CYP2D6 phenotype-related PBPK models for codeine and its active metabolite morphine, which can predict codeine and morphine plasma concentration–time profiles. The model focused on CYP2D6 phenotype-related metabolism and has been used to explore the dosing safety of codeine for different CYP2D6 phenotypes. This study expands the knowledge of CYP2D6 drug–gene interactions and lays the foundation for future exploration of CYP2D6 DDI scenarios.

## Data Availability

The original contributions presented in the study are included in the article/Supplementary Material; further inquiries can be directed to the corresponding author.
