# Supplementary material for: Physiologically based pharmacokinetic modeling to predict the pharmacokinetics of codeine in different CYP2D6 phenotypes
Source: Front Pharmacol. 2024 May 2;15:1342515. doi: 10.3389/fphar.2024.1342515 (PMC11096448; doi:10.3389/fphar.2024.1342515)
Supplement: Supplementary file 1 [file Table1.pdf]

Table S1. Summary of observed PK data of codeine used in PBPK modeling

Source	Ehnic (Phenotype)	Age (years)	N	% Males	Scheme	Dose
Persson 1992[1]	European	52	12	25	iv single dose	20 mg
					Tablet, po single dose	50 mg
Findlay 1986[2]	White American	30.5	4	100	Solution, po single dose	15 mg
						30 mg
						60 mg
Findlay 1986[2]	White American	29.8	6	100	Solution, po single dose	60 mg
Weingarten 1995[3]	White American	/	/	100	Solution, po single dose	100 mg
Dittrich 2011[4]	European	25	16	100	Solution, po qh4, 3 days	23.2 mg
					CR Solution , po q12h, 3 days	69.7 mg
Chen 1989[5]	White American	27	1	0	Tablet, po single dose	30 mg
Wu 2013[6]	Asian-Chinese	22.5	10	100	Tablet, po single dose	30 mg
Yue 1991[7]	Asian	34.4	8	62.5	Tablet, po single dose	50 mg
	Caucasian	33.4	8	100		
Lafolie 1996[8]	European	39	13	77	Tablet, po single dose	50 mg
Quiding 1986[9]	White American	30	12	100	Tablet, po single dose	60 mg
					Tablet, po q8h, 3 days	60 mg
Guay 1987[10]	White American	27.8	6	100	Tablet, po single dose	51.6 mg

					Tablet, po q6h, 3 days	51.6 mg
Shah 1990[11]	White American	29	10	100	Tablet, po single dose	42.4 mg
Mohammed 1993[12]	White American	23	5	100	Tablet, po single dose	60 mg
Ammon 2002[13]	European	31	12	100	Tablet, po single dose	100 mg
Band 1994[14]	European	36.5	13	33	SR Tablet, po single dose	150 mg
					SR Tablet, po q12h, 3 days	150 mg
					IR Tablet, po single dose	44.2 mg
Kim 2002[15]	African American	34.8	19	63	Capsule, po single dose	60 mg
						120 mg
Chen 1991[16]	Caucasian (EM/PM)	27.3<br>25-37	8	88	Tablet, po single dose	30 mg
					Tablet, po q8h, 3 days	30 mg
Tseng 1996[17]	Asian-Chinese (EM/IM/PM)	23.4	32	100	Tablet, po single dose	22.6 mg
Kirchheiner 2007[18]	Caucasian (EM/PM/UM)	18-65	26	100	Tablet, po single dose	30 mg
Wu 2014[19]	Asian-Chinese (EM/PM)	20-24	29	51.7	Tablet, po single dose	30 mg
Yue 1991[20]	Caucasian (EM/PM)	33.4	14	100	Tablet, po single dose	36.8 mg
Mikus 1997[21]	European (EM/PM)	25	10	100	Tablet, po single dose	60 mg
Kronstrand 2001[22]	European (EM)	20	9	33	Tablet, po single dose	100 mg
Eckhardt 1998[23]	European (EM/PM)	32.1	18	55.5	Tablet, po single dose	170 mg

Immedia te-release (IR) tablets, sustained-release (SR) tablets, control-release (CR) solutions

EM: extensive metabolizer, IM: intermediate metabolize, PM: poor metabolizer, UM: ultra-rapid metabolizer.

Table S2. The activity score of different CYP2D6 genotyping

Activity score	Gene	Phenotype
0	*3，*4，*4xN, *5，*6，*7，*16，*36，*40，*42，*56B	PM
0.5-1.0	*9，*10，*17，*29，*41，*45，*46	IM
1.0-2.0	*1，*2，*35，*43，*45xN	EM
>2.0	*1xN,*2xN，*35xN	UM

EM: extensive metabolizer, IM: intermediate metabolize, PM: poor metabolizer, UM: ultra-rapid metabolizer.

Table S3. MFE and GMFE of PK parameters in healthy adult（Mean）

Drugs	Parameter	MFE	GMFE
codeine	$AUC_{0-\infty}$ (mg·h/L)	1.23	1.22
	C_{max} (mg/L)	1.24	1.26
morphine	$AUC_{0-\infty}$ (mg·h/L)	1.52	1.38
	C_{max} (mg/L)	1.17	1.11

MFE: mean fold error; GMFE: geometric mean fold error.

Table S4-1. Accuracy in the estimation of the AUC and C_{max} of codeine

Population	Dose	Method	$AUC_{0-\infty}$ (ng·h/mL)	C_{max} (ng/mL)	t_{max} (h)	$T_{1/2}$ (h)	Source
	20 mg iv	Predicted	155.16	126.72	0.05	9.26	Persson 1992
		Observed	155.16	84.55	0.11	6.27	
		MFE	1.00	1.50	0.45	1.48	
	**Adult iv MFE**		1.00	1.50	0.45	1.48	
	T 30 mg po	Predicted	299.35	77.73	0.70	10.1	Chen 1989
		Observed	331.96	112.04	0.50	3.62	
		MFE	0.90	0.69	1.40	2.79	
		Predicted	276.01	59.37	0.75	8.41	Wu 2013
		Observed	167.40	32.65	1.01	3.49	
		MFE	1.65	1.82	0.74	2.41	
	T 42.4 mg po	Predicted	445.24	104.11	0.70	3.65	Shah 1990
		Observed	325.20	88.10	1.20	2.24	
		MFE	1.37	1.18	0.58	1.63	
	T 50 mg po	Predicted	381.62	79.99	0.75	6.67	Yue 1991

	Observed	358.13	81.02	1.53	2.34	
	MFE	1.07	0.99	0.49	2.85	
	Predicted	584.94	132.42	0.75	8.27	Yue 1991
	Observed	496.94	133.36	1.06	2.94	
	MFE	1.18	0.99	0.71	2.81	
	Predicted	381.62	79.99	0.75	6.67	Persson 1992
	Observed	156.13	37.56	1.56	2.35	
	MFE	2.44	2.13	0.48	2.84	
	Predicted	381.62	79.99	0.75	6.67	Lafolie 1996
	Observed	391.09	86.71	1.15	3.71	
	MFE	0.98	0.92	0.65	1.80	
T 51.6 mg po	Predicted	444.18	104.02	0.70	9.77	Guay 1987
	Observed	649.00	179.30	0.60	4.5	
	MFE	0.68	0.58	1.17	2.17	
T 60 mg po	Predicted	633.10	147.61	0.70	2.04	Quiding 1986
	Observed	630.05	166.49	0.75	2.8	
	MFE	1.00	0.89	0.93	0.73	

	Predicted	633.10	147.61	0.70	2.04	Mohammed 1993
	Observed	779.00	225.00	1.40	2.5	
	MFE	0.81	0.66	0.50	0.82	
T 51.6 mg po q6h	Predicted	630.05	166.49	1.75	9.1	Guay 1987
	Observed	855.00	187.60	1.30	5.6	
	MFE	0.74	0.89	1.35	1.63	
T 60 mg po q8h	Predicted	462.82	135.62	0.75	5.22	Quiding 1986
	Observed	473.00	149.00	1.00	2.5	
	MFE	0.98	0.91	0.75	2.09	
T 100 mg po	Predicted	991.36	207.00	0.75	7.52	Ammon 2002
	Observed	723.00	228.00	0.69	3.37	
	MFE	1.37	0.91	1.09	2.23	
SRT 150 mg po	Predicted	1663.40	183.90	2.50	6.34	Band 1994
	Observed	1202.30	217.00	2.30	2.8	
	MFE	1.38	0.85	1.09	2.26	
SRT 150 mg po q12h	Predicted	2338.59	280.63	3.50	/	Band 1994
	Observed	1576.40	263.80	3.20	/	

	MFE	1.48	1.06	1.09	/	
IRT 44.2 mg po	Predicted	505.52	118.66	0.60	2.71	Band 1994
	Observed	295.35	101.82	0.98	2.6	
	MFE	1.71	1.17	0.61	1.04	
C 60 mg po	Predicted	597.50	139.20	0.70	5.24	Kim 2002
	Observed	734.00	214.20	1.30	2.1	
	MFE	0.81	0.65	0.54	2.50	
C 120 mg po	Predicted	1196.00	279.27	0.70	5.23	Kim 2002
	Observed	1800.00	474.30	1.20	2.4	
	MFE	0.66	0.59	0.58	2.18	
S 60 mg po	Predicted	633.68	151.45	0.60	5.41	Findlay 1986
	Observed	870.00	198.00	1.10	2.1	
	MFE	0.73	0.76	0.55	2.58	
S 100 mg po	Predicted	808.54	130.64	1.00	5.41	Weingarten 1995
	Observed	802.61	167.02	1.00	2.48	
	MFE	1.01	0.78	1.00	2.18	
S 23.2 mg po q4h	Predicted	188.69	84.44	0.60	2.89	Dittrich 2011

		Observed	231.33	79.00	1.20	3.16	
		MFE	0.82	1.07	0.50	0.91	
	CRS 67.6 mg po q12h	Predicted	607.08	85.82	2.75	8.71	Dittrich 2011
		Observed	697.00	96.00	2.90	8.2	
		MFE	0.87	0.89	0.95	1.06	
	**Adult po MFE**		1.12	0.97	0.81	1.98	
	**Adult GMFE**		1.29	1.29	1.47	1.91	
Healthy adult (CYP2D6 EM)	T 22.6 mg po	Predicted	253.98	54.19	1.15	7.75	Tseng 1996
		Observed	280.80	72.16	1.14	2.3	
		MFE	0.90	0.75	1.01	3.37	
	T 30 mg po	Predicted	200.91	45.36	0.75	8.48	Chen 1991
		Observed	243.42	50.55	1.04	3.24	
		MFE	0.83	0.90	0.72	2.62	
		Predicted	200.91	45.36	0.75	8.48	Kirchheiner 2007
		Observed	191	51	/	3.6	
		MFE	1.05	0.89	/	2.36	
		Predicted	276.91	58.43	0.75	8.41	Wu 2014

		Observed	183	39.3	1.25	3.68	
		MFE	1.51	1.49	0.60	2.29	
	T 30 mg po q8h	Predicted	278.56	64.75	0.75	4.45	Chen 1991
		Observed	263.86	75.14	0.86	2.9	
		MFE	1.06	0.86	0.87	1.53	
	T 36.8 mg po	Predicted	240.77	52.57	0.75	4.35	Yue 1991
		Observed	302.35	87.41	1.0	2.43	
		MFE	0.80	0.60	0.75	1.79	
	T 60 mg po	Predicted	596.51	124.5	0.75	7.53	Mikus 1997
		Observed	431.08	198.78	0.54	2.00	
		MFE	1.38	0.63	1.39	3.77	
	T 100 mg po	Predicted	1073.6	228.97	0.9	5.02	Kronstrand 2001
		Observed	772	183	1	2.45	
		MFE	1.39	1.25	0.90	2.05	
	**CYP2D6 EM po MFE**		1.12	0.92	0.89	2.46	
	**CYP2D6 EM GMFE**		1.22	1.30	1.26	2.37	
Healthy adult	T 22.6 mg po	Predicted	256.31	54.41	1.15	7.61	Tseng 1996

(CYP2D6 IM)		Observed	289.18	78.73	1.09	2.4	
		MFE	0.89	0.69	1.06	3.17	
	T 30 mg po	Predicted	277.69	58.49	0.75	8.42	Wu 2014
		Observed	199	41.9	1.28	3.89	
		MFE	1.40	1.40	0.59	2.16	
	**CYP2D6 IM po MFE**		1.14	1.04	0.82	2.67	
	**CYP2D6 IM GMFE**		1.25	1.42	1.34	2.62	
Healthy adult (CYP2D6 PM)	T 22.6 mg po	Predicted	257.68	54.53	1.15	7.62	Tseng 1996
		Observed	298.76	83.82	0.91	2.5	
		MFE	0.86	0.65	1.26	3.05	
	T 30 mg po	Predicted	202.29	45.47	0.75	8.51	Kirchheiner 2007
		Observed	180	45	/	4.8	
		MFE	1.12	1.01	/	1.77	
	T 36.8 mg po	Predicted	241.82	52.65	0.75	5.97	Yue 1991
		Observed	305.35	84.42	0.86	2.9	
		MFE	0.79	0.62	0.87	2.06	
	T 60 mg po	Predicted	599.73	124.77	0.75	7.55	Mikus 199

		Observed	400.54	167.04	0.54	2.52	
		MFE	1.50	0.75	1.39	3.00	
	**CYP2D6 PM po MFE**		1.07	0.76	1.17	2.47	
	**CYP2D6 PM GMFE**		1.21	1.34	1.24	2.40	
Healthy adult (CYP2D6 UM)	T 30 mg po	Predicted	200.49	45.32	0.75	8.48	Kirchheiner 2007
		Observed	192	43	/	3.7	
		MFE	1.04	1.05	/	2.29	
		Predicted	275.23	58.31	0.75	8.39	Wu 2014
		Observed	178	44.3	0.85	3.45	
		MFE	1.55	1.32	0.88	2.43	
	**CYP2D6 UM po MFE**		1.30	1.19	0.88	2.36	
	**CYP2D6 UM GMFE**		1.27	1.18	1.13	2.36	

C: Capsule; T: tablets; SR: sustained-release (SR) tablets; CRS: control-release (CR) solutions; IRT: Immediate-release (IR) tablets

EM: extensive metabolizer, IM: intermediate metabolize, PM: poor metabolizer, UM: ultra-rapid metabolizer.

MFE: mean fold error; GMFE: geometric mean fold error.

Table S4-2. Accuracy in the estimation of the AUC and C_{max} of morphine

Population	Dose	Method	$AUC_{0-\infty}$ (ng·h/mL)	C_{max} (ng/mL)	t_{max} (h)	$T_{1/2}$ (h)	Source
Healthy adults	T 30 mg po	Predicted	8.92	2.13	1.05	6.65	Wu 2013
		Observed	5.38	1.48	0.99	5.34	
		MFE	1.66	1.44	1.06	1.25	
	T 42.2 mg po	Predicted	12.72	2.97	1.0	/	Shah 1990
		Observed	10.9	2.7	1.2	/	
		MFE	1.17	1.10	0.83	/	
	T 50 mg po	Predicted	8.65	2.02	1.05	7.11	Yue 1991
		Observed	6.29	2.25	1.04	2.72	
		MFE	1.38	0.90	1.01	2.61	
		Predicted	8.65	2.02	1.05	4.6	Lafolie 1996
		Observed	7.06	2.17	1.0	8.27	
		MFE	1.23	0.93	1.05	0.56	
	T 51.6 mg po	Predicted	17.10	4.15	0.70	/	Guay 1987
		Observed	69.26	9.1	0.75	/	

	MFE	0.25	0.46	0.93	/	
T 60 mg po	Predicted	18.03	4.18	1	4.93	Quiding 1986
	Observed	7.11	2.4	1.1	4.2	
	MFE	2.54	1.74	0.91	1.17	
T 60 mg po q8h	Predicted	14.76	3.95	1.0	5.05	Quiding 1986
	Observed	12.4	3.8	1.0	4.2	
	MFE	1.19	1.04	1.00	1.20	
T 100 mg po	Predicted	42.11	8.68	1.10	6.94	Ammon 2002
	Observed	26.96	6.39	0.63	8.78	
	MFE	1.56	1.36	1.75	0.79	
S 15mg po	Predicted	5.3	1.19	0.95	/	Findlay 1986
	Observed	8.83	1.57	1.49	/	
	MFE	0.60	0.76	0.64	/	
S 30mg po	Predicted	10.57	2.35	0.95	/	Findlay 1986
	Observed	14.78	3.69	0.48	/	
	MFE	0.72	0.64	1.98	/	
S 60mg po	Predicted	18.04	4.22	0.90	/	Findlay 1986

		Observed	25.54	7.42	1.0	/	
		MFE	0.71	0.57	0.90	/	
	**Adult po MFE**		1.18	0.99	1.10	1.26	
	**Adult GMFE**		1.62	1.39	1.23	1.48	
Healthy adult (CYP2D6 EM)	T 30 mg po	Predicted	6.06	1.5	1.05	6.39	Kirchheiner 2007
		Observed	7.44	1.76	/	13	
		MFE	0.81	0.85	/	0.49	
		Predicted	5.37	1.28	1.05	6.64	Wu 2014
		Observed	5.05	0.96	0.86	11.5	
		MFE	1.06	1.33	1.22	0.58	
	T 36.8 mg po	Predicted	5.27	1.12	1.1	2.73	Yue 1991
		Observed	3.98	1.80	1.0	5.7	
		MFE	1.32	0.62	1.10	0.48	
	T 60 mg po	Predicted	15.36	3.17	1.1	6.94	Mikus 1997
		Observed	7.93	3.97	0.54	3.6	
		MFE	1.94	0.80	2.04	1.93	
	T 100 mg po	Predicted	28.77	6.31	1.25	3.16	Kronstrand 2001

		Observed	34	5.9	1	3.48	
		MFE	0.85	1.07	1.25	0.91	
	T 170 mg po	Predicted	37.99	8.32	1.25	7.02	Eckhardt 1998
		Observed	49.39	10.90	/	4.5	
		MFE	0.77	0.76	/	1.56	
	**CYP2D6 EM po MFE**		1.13	0.91	1.40	0.99	
	**CYP2D6 EM GMFE**		1.31	1.28	1.36	1.70	
Healthy adult (CYP2D6 IM)	T 30 mg po	Predicted	2.24	0.53	1.05	6.64	Wu 2014
		Observed	3.26	0.68	0.86	6.84	
		Predicted	0.69	0.78	1.22	0.97	
	**CYP2D6 IM po MFE**		0.69	0.78	1.22	0.97	
Healthy adult (CYP2D6 PM)	T 30 mg po	Predicted	0.08	0.02	1.05	6.41	Kirchheiner 2007
		Observed	0.09	0.03	/	17	
		MFE	0.89	0.67	/	0.38	
	T 60 mg po	Predicted	0.49	0.10	1.1	6.95	Mikus 1997
		Observed	0.54	0.19	0.62	4.8	
		MFE	0.91	0.53	1.77	1.45	

	T 170 mg po	Predicted	1.2	0.26	1.25	7.03	Eckhardt 1998
		Observed	2.94	0.57	/	5.00	
		MFE	0.41	0.46	/	1.41	
	**CYP2D6 PM po MFE**		0.73	0.55	1.77	1.08	
	**CYP2D6 PM GMFE**		1.45	1.84	1.77	1.75	
Healthy adult (CYP2D6 UM)	T 30 mg po	Predicted	7.83	1.94	1.05	6.39	Kirchheiner 2007
		Observed	10.12	1.92	/	14.00	
		MFE	0.77	1.01	/	0.46	
		Predicted	12.01	2.88	1.05	6.62	Wu 2014
		Observed	8.52	2.06	0.64	9.4	
		MFE	1.41	1.40	1.64	0.70	
	**CYP2D6 UM po MFE**		1.09	1.20	1.64	0.58	
	**CYP2D6 UM GMFE**		1.35	1.19	1.64	1.76	

C: Capsule; T: tablets; S: solutions;

EM: extensive metabolizer, IM: intermediate metabolize, PM: poor metabolizer, UM: ultra-rapid metabolizer.

MFE: mean fold error; GMFE: geometric mean fold error.

Table S4-3. Accuracy in the estimation of the AUC and C_{max} of unconjugated codeine

Population	Dose	Method	$AUC_{0-\infty}$ (ng·h/mL)	C_{max} (ng/mL)	t_{max} (h)	Source
Healthy adults	S 15mg po	Predicted	133	31.01	0.6	Findlay 1986
		Observed	106	32	0.6	
		MFE	1.25	0.97	1.00	
	S 30mg po	Predicted	266.26	62.04	0.6	Findlay 1986
		Observed	216	61	0.8	
		MFE	1.23	1.02	0.75	
	S 60mg po	Predicted	633.68	151.45	0.6	Findlay 1986
		Observed	439	114	1.1	
		MFE	1.44	1.33	0.55	
	Adult po MFE		1.31	1.10	0.77	
	Adult GMFE		1.31	1.11	1.34	

MFE: mean fold error; GMFE: geometric mean fold error.

Table S5. Accuracy of codeine and morphine in the estimation of single-dose(30mg) models

CYP2D6	Predicted		Observed		Accuracy	
Phenotype	$AUC_{0-\infty}$ (mg·h/L)	C_{max} (mg/L)	$AUC_{0-\infty}$ (mg·h/L)	C_{max} (mg/L)	AUC (%)	C max (%)
**Codeine**						
PM	202.29	45.47	180	45	12.38	1.04
IM	277.69	58.49	199	41.9	39.54	39.59
EM	226.24	49.72	205.81	46.95	9.93	5.89
UM	237.86	51.82	185.00	43.65	28.57	18.71
**Morphine**						
PM	0.08	0.02	0.09	0.03	11.11	33.33
IM	2.24	0.53	3.26	0.68	31.29	22.06
EM	5.72	1.39	6.25	1.36	8.49	2.21
UM	9.92	2.41	9.32	1.99	6.44	21.11

EM: extensive metabolizer, IM: intermediate metabolize, PM: poor metabolizer, UM: ultra-rapid metabolizer.

References:

[1]. Persson, K., et al., The postoperative pharmacokinetics of codeine. Eur J Clin Pharmacol, 1992. 42(6): p. 663-6.

[2]. Findlay, J.W., et al., Comparative disposition of codeine and pholcodine in man after single oral doses. Br J Clin Pharmacol, 1986. 22(1): p. 61-71.

[3]. Weingarten, B., H.Y. Wang and D.M. Roberts, Determination of codeine in human plasma by high-performance liquid chromatography with fluorescence detection. J Chromatogr A, 1995. 696(1): p. 83-92.

[4]. Dittrich, P., et al., Pharmacokinetics of a novel liquid controlled release codeine formulation. Drug Dev Ind Pharm, 2011. 37(9): p. 1119-24.

[5]. Chen, Z.R., et al., Direct determination of codeine-6-glucuronide in plasma and urine using solid-phase extraction and high-performance liquid chromatography with fluorescence detection. J Chromatogr, 1989. 493(2): p. 313-24.

[6]. Wu, X., et al., Simultaneous analysis of codeine and its active metabolites in human plasma using liquid chromatography-tandem mass spectrometry: application to a pharmacokinetic study after oral administration of codeine. J Pharm Biomed Anal, 2013. 78-79: p. 261-8.

[7]. Yue, Q.Y., et al., A comparison of the pharmacokinetics of codeine and its metabolites in healthy Chinese and Caucasian extensive hydroxylators of debrisoquine. Br J Clin Pharmacol, 1991. 31(6): p. 643-7.

[8]. Lafolie, P., et al., Urine and plasma pharmacokinetics of codeine in healthy volunteers: implications for drugs-of-abuse testing. J Anal Toxicol, 1996. 20(7): p. 541-6.

[9]. Quiding, H., et al., Plasma concentrations of codeine and its metabolite, morphine, after single and repeated oral administration. Eur J Clin Pharmacol, 1986. 30(6): p. 673-7.

[10]. Guay, D.R., et al., Pharmacokinetics of codeine after single- and multiple-oral-dose administration to normal volunteers. J Clin Pharmacol, 1987. 27(12): p. 983-7.

[11]. Shah, J.C. and W.D. Mason, Plasma codeine and morphine concentrations after a single oral dose of codeine phosphate. J Clin Pharmacol, 1990. 30(8): p. 764-6.

[12]. Mohammed, S.S., et al., Codeine disposition in sickle cell patients compared with healthy volunteers. J Clin Pharmacol, 1993. 33(9): p. 811-5.

[13]. Ammon, S., et al., Diclofenac does not interact with codeine metabolism in vivo: a study in healthy volunteers. BMC Clin Pharmacol, 2002. 2: p. 2.

[14]. Band, C.J., et al., Human pharmacokinetic study of immediate-release (codeine phosphate) and sustained-release (codeine Contin) codeine. J Clin Pharmacol, 1994. 34(9): p. 938-43.

[15]. Kim, I., et al., Plasma and oral fluid pharmacokinetics and pharmacodynamics after oral codeine administration. Clin Chem, 2002. 48(9): p. 1486-96.

[16]. Chen, Z.R., et al., Disposition and metabolism of codeine after single and chronic doses in one poor and seven extensive metabolisers. Br J Clin Pharmacol, 1991. 31(4): p. 381-90.

[17]. Tseng, C.Y., et al., Formation of morphine from codeine in Chinese subjects of different CYP2D6 genotypes. Clin Pharmacol Ther, 1996. 60(2): p. 177-82.

[18]. Kirchheiner, J., et al., Pharmacokinetics of codeine and its metabolite morphine in ultra-rapid metabolizers due to CYP2D6 duplication. Pharmacogenomics J, 2007. 7(4): p. 257-65.

[19]. Wu, X., et al., The impact of CYP2D6 polymorphisms on the pharmacokinetics of codeine and its metabolites in Mongolian Chinese subjects. Eur J Clin Pharmacol, 2014. 70(1): p. 57-63.

[20]. Yue, Q.Y., et al., Pharmacokinetics of codeine and its metabolites in Caucasian healthy volunteers: comparisons between extensive and poor hydroxylators of debrisoquine. Br J Clin Pharmacol, 1991. 31(6): p. 635-42.

[21]. Mikus, G., et al., Effect of codeine on gastrointestinal motility in relation to CYP2D6 phenotype. Clin Pharmacol Ther, 1997. 61(4): p. 459-66.

[22]. Kronstrand, R. and A.W. Jones, Concentration ratios of codeine-to-morphine in plasma after a single oral dose (100 mg) of codeine phosphate. J Anal Toxicol, 2001. 25(6): p. 486-7.

[23]. Eckhardt, K., et al., Same incidence of adverse drug events after codeine administration irrespective of the genetically determined differences in morphine formation. Pain, 1998. 76(1-2): p. 27-33.
